# HSCVFNT: Inference of Time-Delayed Gene Regulatory Network Based on Complex-Valued Flexible Neural Tree Model

**DOI:** 10.3390/ijms19103178

**Published:** 2018-10-15

**Authors:** Bin Yang, Yuehui Chen, Wei Zhang, Jiaguo Lv, Wenzheng Bao, De-Shuang Huang

**Affiliations:** 1 School of Information Science and Engineering, Zaozhuang University, Zaozhuang 277100, China; batsi@126.com (B.Y.); batsi_china@hotmail.com (W.Z.); 1531680@tongji.edu.cn (J.L.); 2 School of Information Science and Engineering, University of Jinan, Jinan 250002, China; yhchen@ujn.edu.cn; 3 School of Computer Science, China University of Mining and Technology, Xuzhou 221000, China; 4 Institute of Machine Learning and Systems Biology, Tongji University, Shanghai 200092, China; 1510479@tongji.edu.cn

**Keywords:** time-delayed, reducing redundancy methods, information theory, flexible neural tree

## Abstract

Gene regulatory network (GRN) inference can understand the growth and development of animals and plants, and reveal the mystery of biology. Many computational approaches have been proposed to infer GRN. However, these inference approaches have hardly met the need of modeling, and the reducing redundancy methods based on individual information theory method have bad universality and stability. To overcome the limitations and shortcomings, this thesis proposes a novel algorithm, named HSCVFNT, to infer gene regulatory network with time-delayed regulations by utilizing a hybrid scoring method and complex-valued flexible neural network (CVFNT). The regulations of each target gene can be obtained by iteratively performing HSCVFNT. For each target gene, the HSCVFNT algorithm utilizes a novel scoring method based on time-delayed mutual information (TDMI), time-delayed maximum information coefficient (TDMIC) and time-delayed correlation coefficient (TDCC), to reduce the redundancy of regulatory relationships and obtain the candidate regulatory factor set. Then, the TDCC method is utilized to create time-delayed gene expression time-series matrix. Finally, a complex-valued flexible neural tree model is proposed to infer the time-delayed regulations of each target gene with the time-delayed time-series matrix. Three real time-series expression datasets from (Save Our Soul) SOS DNA repair system in *E. coli* and *Saccharomyces cerevisiae* are utilized to evaluate the performance of the HSCVFNT algorithm. As a result, HSCVFNT obtains outstanding F-scores of 0.923, 0.8 and 0.625 for SOS network and (In vivo Reverse-Engineering and Modeling Assessment) IRMA network inference, respectively, which are 5.5%, 14.3% and 72.2% higher than the best performance of other state-of-the-art GRN inference methods and time-delayed methods.

## 1. Introduction

Gene regulatory networks (GRN) contain the regulation relationships among genes, mRNA and proteins, which almost control all biological activity in the field of biology [1,2,3]. Mastering the gene regulation mechanism could help understand the growth and development of animals and plants, and master a key of revealing the mystery of biology [4,5,6,7,8]. The gene regulatory network can be regarded as a complex dynamics system, which has some characteristics, including strong coupling, random, time-delayed, and strongly nonlinear [9,10,11,12]. In addition, the number of genes of gene expression data is far more than the number of sample points. To infer a gene regulatory network accurately from time-series gene expression data is a challenging task during several decades [13,14]. 

The gene regulation pattern in organisms involves a time-delayed factor [15,16,17,18]. At present, many GRN inference methods related to time-delayed factor have been proposed to identify GRN. Li et al. developed a new method that combined relative change ratio and time-delayed dynamic Bayesian network (TDBN) to infer networks [19]. Chueh et al developed a new approach to reconstruct time-delayed Boolean networks as a tool for exploring biological pathways [20]. Zoppoli proposed TimeDelay-ARACNE to identify time-delayed dependencies between the expression profiles by assuming as an underlying probabilistic model a stationary Markov Random Field [21]. Chowdhury et al. proposed a novel Time-Delayed S-System (TDSS) model to infer both instantaneous and time-delayed interactions of GRN [22]. Mundra et al. proposed a two-step method based on cross-correlation and LASSO (TDLASSO) to infer time-delayed network [23]. Li et al. proposed a new approach, named Max-Min high-order dynamic Bayesian network (MMHO-DBN) for inferring time-delayed regulations in GRN [24]. Kordmahalleh et al. presented a hierarchical recurrent neural network (HRNN) to infer time-delayed gene interactions [25].

In order to improve the accuracy of GRN inference, many reducing redundancy methods are utilized before or after GRN identification by the inference methods. Generally, the information theoretic approaches were utilized to measure the complex regulatory dependence between genes [26]. Zhang et al. presented NARROMI to improve the accuracy of GRN, in which noisy and redundant regulations were first removed using mutual information (MI) and recursive optimization (RO) [27]. Liu et al. proposed a novel algorithm, namely local Bayesian network (LBN), which utilized conditional mutual information (CMI) to reduce redundant regulations [28]. Because MI does not work well for continuous multivariate variables, Akhand et al. incorporated maximal information coefficient (MIC) into minimal redundancy network for GRN inference [29]. Liu et al. proposed a novel minimum-redundancy network (MRNET) algorithm to improve regulatory network structures [30]. These existing methods have two disadvantages: (1) MI could detect nonlinear dependencies of two variables, but it is not fit for continuous multivariate variables. MIC could quickly evaluate the relationship of the variables with different functions (linear, exponential and periodic). However, when the null hypothesis could not be established, the statistics ability of MIC will be affected. The individual method has their strengths and weaknesses; (2) the existing reducing redundancy methods define a threshold, and the edges whose weights are less than the threshold will be removed. Because the dependence degrees between each target gene and its regulatory factors are not necessarily on one level, a unified threshold could delete some true positive edges and suffer from false positive problem.

To overcome these limitations and shortcomings of these existing methods, this paper proposes a novel algorithm, namely HSCVFNT (the website is http://121.250.173.184), to infer a gene regulatory network with time-delayed regulations by utilizing a hybrid scoring method and complex-valued flexible neural network (CVFNT). In order to reduce computation complexity, a decomposed strategy is utilized. A HSCVFNT algorithm infers the regulations of each target gene, respectively. For each target gene, the HSCVFNT algorithm uses a novel scoring method based on time-delayed mutual information (TDMI), time-delayed maximum information coefficient (TDMIC) and time-delayed correlation coefficient (TDCC), to reduce the redundant regulatory relationships. In the scoring method, the ranks of regulation relationships according to TDMI, TDMIC and TDCC are added. L regulatory factors with the minimal rankings are selected as the candidate regulatory factor set of the target gene. Then, according to the maximum time lag $\tau_{\max}$, TDCC is utilized to search the optimal time lags between target gene and its regulatory factors, and create time-delayed gene expression time-series matrix. 

Compared with real values, complex numbers can have richer representation ability, and also promote the memory retrieval mechanism of noise robust [31]. Complex-valued versions of a large number of models have been presented to solve forecasting and classification problems [32,33,34]. Xiong et al. proposed complex-valued radial basis function neural networks (FCRBFNNs) to predict real interval stock price time series data [35]. Saoud et al. presented a fully complex-valued wavelet network (FCWN) to predict the global solar irradiation [36]. Savitha et al. proposed a Circular Complex-valued Extreme Learning Machine (CC-ELM) to classify an acoustic emission signal and mammogram problem [37]. In this paper, a complex-valued flexible neural tree model is proposed to model a time-delayed matrix in order to infer the time-delayed regulations. The final structure of GRN can be obtained by an iteratively performing HSCVFNT algorithm.

Three real time-series expression datasets from an SOS DNA repair system in *E. coli* and *Saccharomyces cerevisiae* (IRMA network) are utilized to evaluate the performance of HSCVFNT algorithm. Experimental results show that HSCVFNT performs better than other state-of-the-art GRN inference methods.

## 2. Results

### 2.1. Datasets and Evaluation Metrics

Two real-life benchmark gene regulatory networks are utilized to validate our method. The first GRN is derived from the SOS DNA damage repair network of *E. coli* with six genes [38], and the second benchmark network is from *Saccharomyces cerevisiae* (IRMA network), which contains five genes [39].

**Figure 1:**
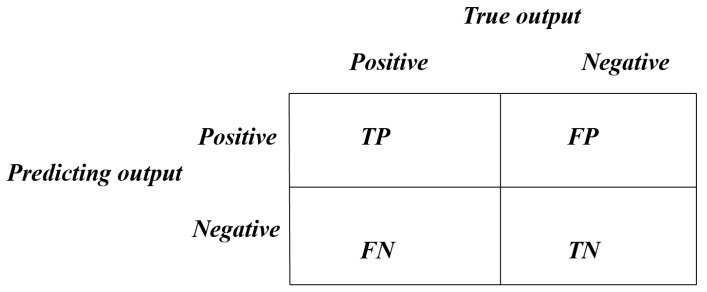
Description of TP, FP, FN and TN.

*Sensitivity*, *Precision*, *Specificity* and F-score are utilized to evaluate the performance of HSCVFNT algorithm. Four criterions are defined as follows:

(1) $$Sensitivity=\frac{TP}{TP+FN},$$

(2) $$Precision=\frac{TP}{TP+FP},$$

(3) $$Specificity=\frac{TN}{FP+TN},$$

(4) $$F-score=2\frac{Sensitivity*Precision}{Sensitivity+Precision},$$

where *TP*, *FP*, *FN*, and *TN* are described in Figure 1.

To evaluate the performance of the HSCVFNT method, the HSCVFNT algorithm is compared with the algorithms with better performance. For the SOS DNA repair network in *E. coli*, we select the algorithms of DBN [40], S-system [41] and TDSS (time-delayed S-system) [22]. For the IRMA network, six algorithms are selected for deriving GRNs, such as TDARACNE (the ARACNE algorithm based on time-delayed mutual information) [21], TDLASSO (a high-order LASSO) [42], DBN-ZC (a high-order DBN) [43], DBmcmc (a first-order DBN) [44], MMHO-DBN [24] and HRNN [25]. For all the methods in the comparison, the parameters are set by default. 

### 2.2. Network Construction of Real Data of E. coli SOSDNA Repair Network

**Figure 2:**
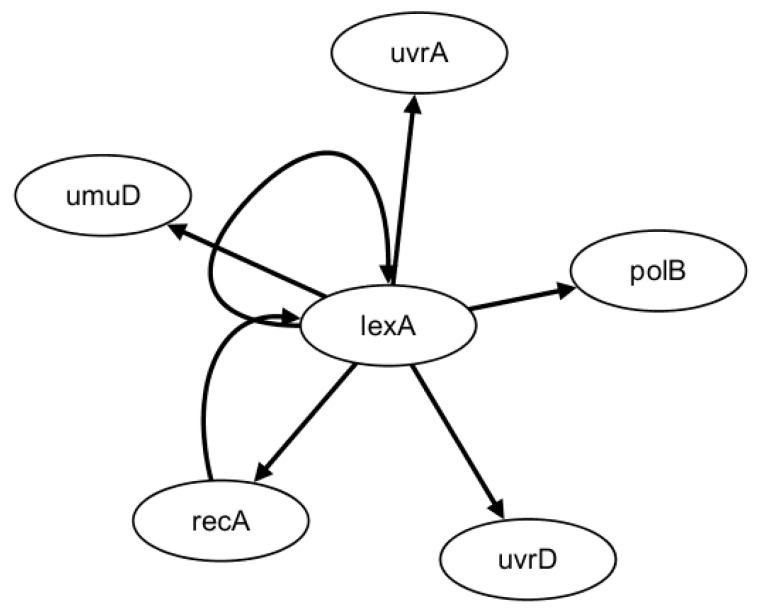
*E. coli* SOS DNA repair network*.*

SOS DNA damage repair network of *E. coli* includes four experiments under different intensities of light (Experiments 1 and 2 at 5 ^Jm2^, Experiments 3 and 4 at 20 ^J m2^) [45]. Each experiment consists of 50 time points and the interval between two time points is six minutes, which involves eight genes: *uvrD*, *lexA*, *umuD*, *recA*, *uvrA*, *uvrY*, *ruvA* and *polB*. In this paper, six main genes (*uvrD*, *lexA*, *umuD*, *recA*, *uvrA* and *polB*) are selected to test our method. The true gene regulatory network is described in Figure 2.

**Figure 3:**
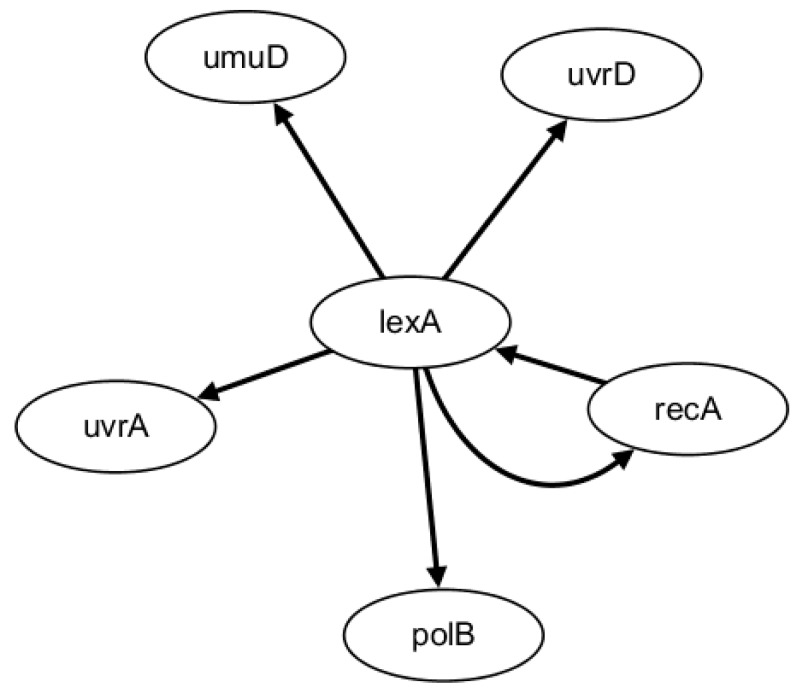
The inferred SOS network.

In the HSCVFNT algorithm, the maximum time lag $\tau_{max}$ is set as 6. The number of candidate regulatory factors L is set as 2. The inferred GRN is depicted in Figure 3. The solid lines represent the true regulatory relationships and the dotted lines are the false positive regulations inferred. Comparing Figure 2 and Figure 3, we can see that our inferred six edges are all true positive and no false positive edges occur. Only edge lexA→lexA could not be inferred. This is because the target gene lexA could not select itself into its candidate regulatory factor set and self-regulation relationships are not considered.

The performance comparison results are shown in Table 1. From Table 1, it can be seen that the Specificity and F-score indicators of the HSCVFNT method are the best of these four methods, which are 1.00 and 0.923, respectively. Sensitivity of our method is 14.3% lower than that of the TDSS method, which reveals that the TDSS method infers more true positive regulations than our method. However, specificity of our method is 11.4% higher than that of the TDSS method, and F-score is 5.5% higher than the TDSS method, which reveal that our method infers less false positive edges. On the whole, the main results of our method are better than other comparison methods. Through 20 runs, Sensitivity, Specificity and F-score obtained from HSCVFNT average about 0.8286 ± 0.0602, 0.9897 ± 0.017 and 0.8856 ± 0.0541, respectively. Compared with other methods in Table 1, HSCVFNT has less Sensitivity than the S-system and TDSS, but it has a convincing Specificity and F-score, which reveals that HSCVFNT could have stable performance for SOS network inference.

### 2.3. Network Construction of Real Data of the IRMA Network

**Figure 4:**
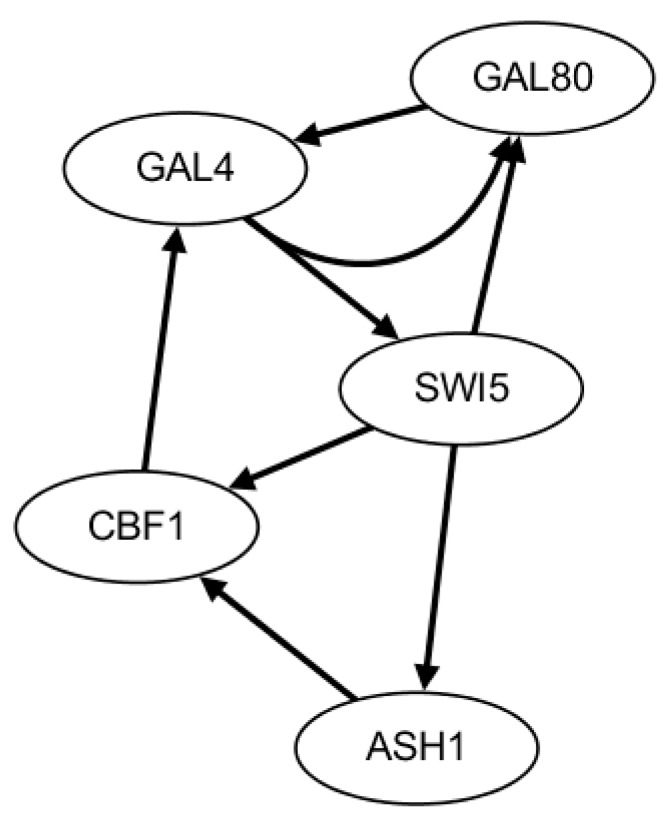
The standard IRMA network.

The second benchmark network is from *Saccharomyces cerevisiae* (IRMA network). Two gene expression time-series datasets are collected with 21 equally distributed time points by being triggered by glucose within the network [46]. In the first dataset, glucose medium is switched to galactose (switched on, named on dataset). In the second dataset, galactose medium is switched to glucose (switched off, named off dataset). The true gene regulatory network is described in Figure 4 containing five genes and eight edges.

**Figure 5:**
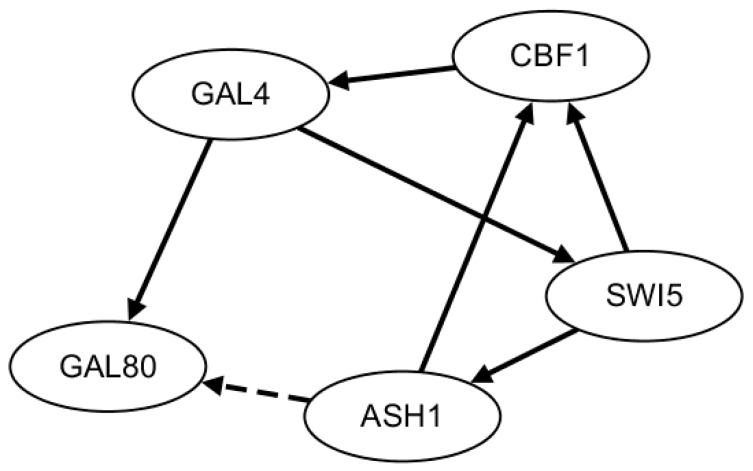
The inferred IRMA network with on dataset. The solid lines represent the true regulatory relationships and the dotted lines are the false positive regulations inferred.

**Figure 6:**
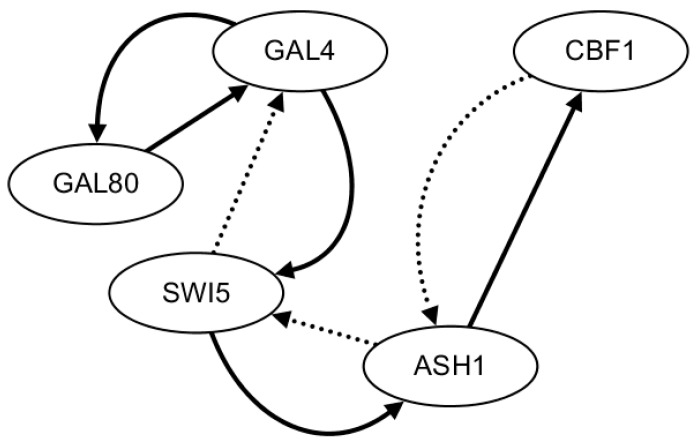
The inferred IRMA network with off dataset. The solid lines represent the true regulatory relationships and the dotted lines are the false positive regulations inferred.

In the HSCVFNT method, the maximum time lag $\tau_{max}$ is set as 6. The number of candidate regulatory factors L is set as 2 for on dataset and off dataset. The inferred GRN with on dataset is depicted in Figure 5. The inferred GRN contains seven edges, of which six edges are true positive and one edge is false positive. The inferred GRN with off dataset is depicted in Figure 6. The inferred GRN contains eight edges, of which six edges are true positive and two edges are false positive. Compared with the golden network, the HSCVFNT algorithm could identify 75% true positive regulations and less false positive edges.

The performance comparison results with on dataset are shown in Table 2. From Table 2, it can be seen that the Sensitivity and F-score indicators of our method are the best of these seven methods, which are 0.75 and 0.8, respectively. Precision of our method is 28.5% higher than HRNN, 14.3% lower than MMHO-DBN, 19.99% higher than TDARACNE, 114% higher than TDLASSO, 71.4% higher than DBmcmc and 42.8% higher than DBN-ZC. In terms of Sensitivity, our method is 50% higher than MMHO-DBN, 20% higher than TDARACNE, 200% higher than TDLASSO, 200% higher than DBmcmc and 100% higher than DBN-ZC. In terms of F-score, our method is 14.3% higher than HRNN, 20% higher than MMHO-DBN, 20% higher than TDARACNE, 166.7% higher than TDLASSO, 140.2% higher than DBmcmc and 73.3% higher than DBN-ZC.

The performance comparison results with off dataset are shown in Table 3. From Table 3, it can be seen that the Sensitivity and F-score two indicators of HSCVFNT algorithm are all the best of these five methods, which are 0.625 and 0.625, respectively. Precision of our method is 0.625, which is 6.25% lower than MMHO-DBN, 25% higher than TDARACNE, 150% higher than TDLASSO, and 267.6% higher than DBmcmc. Sensitivity of our method is 150% higher than MMHO-DBN, 400% higher than TDARACNE, 400% higher than TDLASSO, and 400% higher than DBmcmc. In terms of F-score, our method is 72.2% higher than MMHO-DBN, 212.5% higher than TDARACNE, 274.9% higher than TDLASSO, and 344.2% higher than DBmcmc.

Although the MMHO-DBN algorithm has the highest Precision with on and off datasets, which reveals that the ratio of true positive edges is very high, Sensitivity and F-score are not perfect. MMHO-DBN algorithm only inferred four and one true positive edges with on and off dataset, respectively. F-score of MMHO-DBN is lower than our method. On the whole, our method performs better.

In order to test the stability of HSCVFNT, 20 runs are performed for IRMA network identification with on and off datasets. With on dataset, Precision, Sensitivity and F-score obtained average 0.817 ± 0.064, 0.736 ± 0.042 and 0.774 ± 0.045, respectively. With off dataset, the averaged Precision, Sensitivity and F-score are 0.569 ± 0.066, 0.612 ± 0.04 and 0.589 ± 0.047, respectively. Compared with Table 2 and Table 3, HSCVFNT has the highest F-score on average.

## 3. Discussion

### 3.1. Influence of Time-Delayed Factor

In order to verify the effect of time-delayed factor on the performance of gene regulatory network modeling, we also use an HSCVFNT algorithm with no delay (non time-delayed version) for an SOS network and IRMA network identification. In the non time-delayed version, the scoring method is based on mutual information, maximum information coefficient and correlation coefficient, and time series data are also non time-delayed.

**Figure 7:**
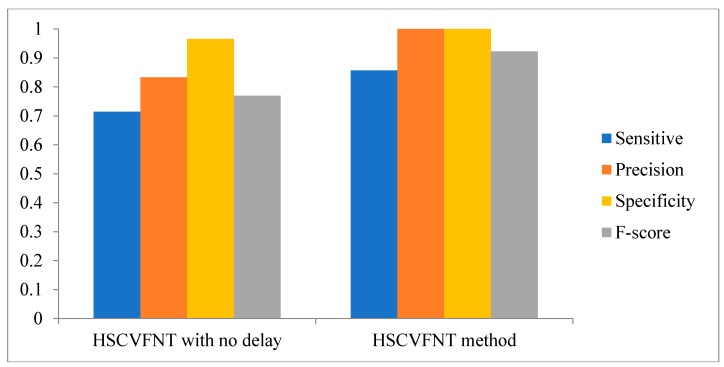
Effect of time-delayed factor for SOS network inference.

**Figure 8:**
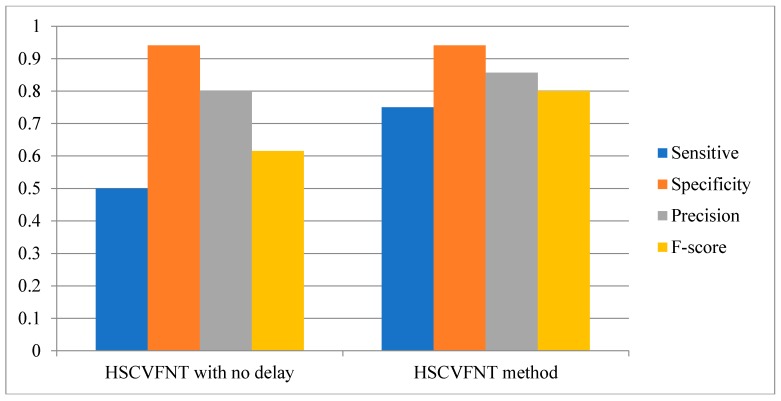
Effect of time-delayed factor for IRMA network inference with on dataset.

**Figure 9:**
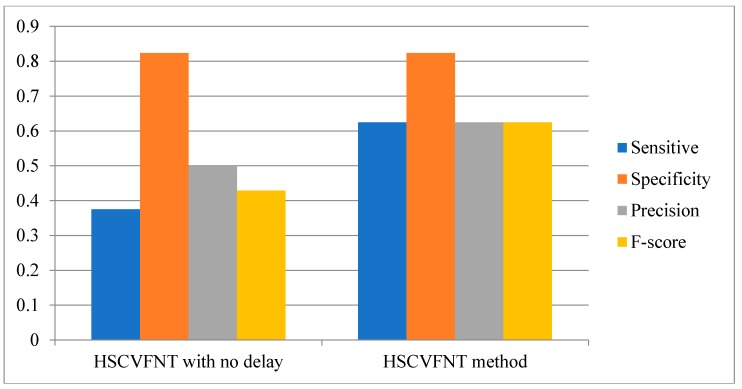
Effect of time-delayed factor for IRMA network inference with off dataset.

Comparison results are depicted in Figure 7, Figure 8 and Figure 9, respectively. The Sensitivity, Specificity, Precision and F-score four indicators of the HSCVFNT method are all higher than HSCVFNT method with no delay. For the construction of an SOS network, the Sensitivity, Specificity, Precision and F-score of our algorithm are 20%, 20.4%, 3.6%, and 20% higher than those of the HSCVFNT method with no delay, respectively. For the construction of IRMA network with on dataset, the Sensitivity, Precision and F-score of our algorithm are 50%, 7.1%, and 30.1% higher than those of our method with no delay, respectively. For the construction of IRMA network with off dataset, the Sensitivity, Precision and F-score of HSCVFNT algorithm are 66.7%, 25%, and 45.7% higher than those of the HSCVFNT method with no delay, respectively. It could demonstrate that the HSCVFNT method with time-delayed factor could identify a gene regulatory network more accurately.

### 3.2. Influence of the Number of Candidate Regulatory Factors L

**Figure 10:**
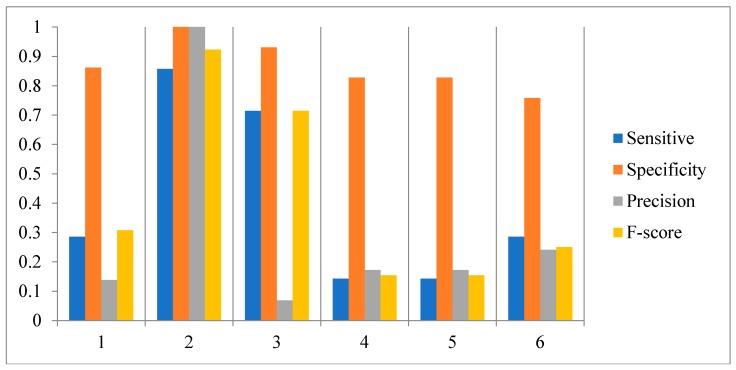
Effect of parameter L for SOS network inference.

**Figure 11:**
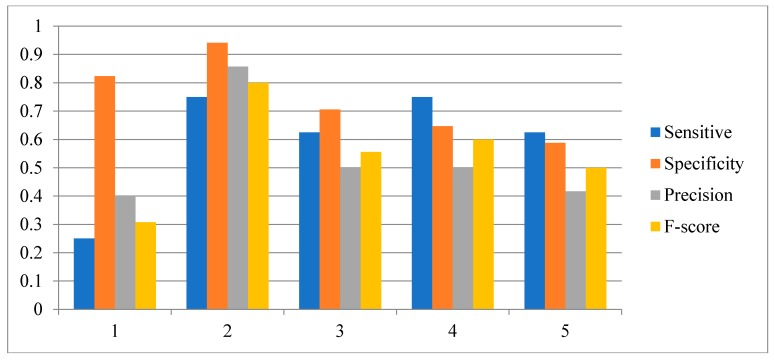
Effect of parameter L for IRMA network inference with on dataset.

**Figure 12:**
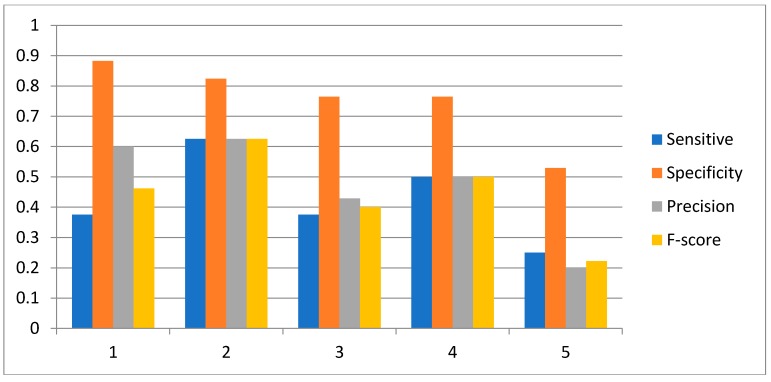
Effect of parameter L for IRMA network inference with off dataset.

In order to investigate the effect of the number of candidate regulatory factors L on the performance of the HSCVFNT method, we make the comparison experiments with different L. For SOS network inference, L is set from 1 to 6. For IRMA network inference, L is set from 1 to 5. The experiment results are described in Figure 10, Figure 11 and Figure 12, respectively. From Figure 10 and Figure 11, it can be seen that when L is set as 2, our method has the highest Sensitivity, Precision, Specificity and F-score. Figure 12 shows that, when L is set as 2, our method has the highest Sensitivity, Precision and F-score. To choose the proper L is very important for the performance of the HSCVFNT algorithm. The performances of different L show that about 30% of the number of genes is more appropriate. 

### 3.3. Performance of Our Proposed Scoring Method

**Figure 13:**
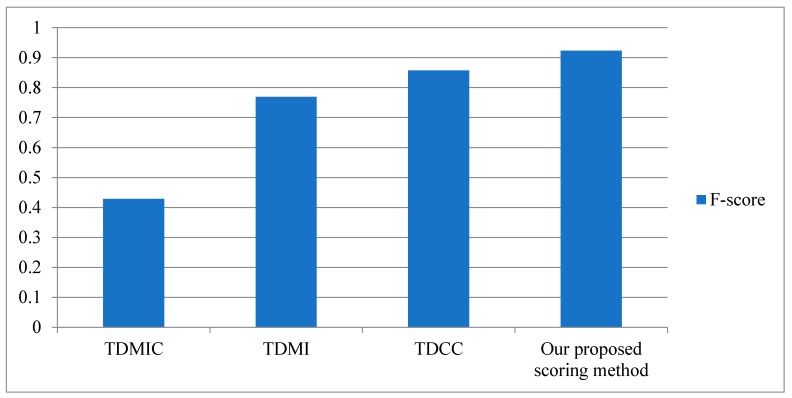
F-score results of four reducing redundancy methods.

In order to validate the performance of our proposed scoring method, we make the comparison experiments with the single time-delayed reducing redundancy methods (TDMIC, TDMI, and TDCC) for inferring an SOS network. F-score results of four reducing redundancy methods are depicted in Figure 13, which reveal that our proposed scoring method has the highest F-score, with a more true positive rate and lower false positive rate.

### 3.4. Performance of Our Selected Stochastic Optimizer

In order to validate the performance of the bat algorithm (BA), we make the comparison experiments with the classical optimization algorithms (genetic algorithm (GA), particle swarm optimization (PSO) and differential evolution (DE)), which are utilized to optimize the CVFNT model. Through 20 runs, performance results of four optimization methods for SOS network and IRMA network inference are listed in Table 4, Table 5 and Table 6, respectively (SD: standard deviation). Hit ratio is the percentage of the best GRNs obtained over all runs. From the results, it can be seen that our selected optimization method has more accurate and robust performance than PSO, DE and GA. 

## 4. Method

### 4.1. Our Proposed Scoring Method

#### 4.1.1. Time-Delayed Mutual Information

Mutual information (MI) between two genes *X* and *Y* is defined as [47,48]

(5) $$M(X,Y)=\sum_{x\in X}\sum_{y\in Y}p(x,y)\log\frac{p(x,y)}{p(x)p(y)},\;$$

where *p*(*x*) and *p*(*y*) are the marginal probability distribution functions of gene *X* and gene *Y*, respectively. *p*(*x*, *y*) is the joint probability density function of gene *X* and gene *Y*. MI is symmetric, so it could not identify the time-delayed dependence between two genes. Time-delayed mutual information (TDMI) is proposed to measure the time-delayed dependence between target gene and its regulatory factors.

The time-delayed mutual information is defined as follows:

(6) $$M^{\tau }(X,Y)=\sum_{k=1}\tilde{P}(X_{}^{k},Y_{}^{k+\tau })log\frac{\tilde{P}(X_{}^{k},Y_{}^{k+\tau })}{\tilde{P}(X_{}^{k})\tilde{P}(Y_{}^{k+\tau })},$$

where $M^{\tau }(X,Y)$ represents the mutual information between gene X and gene Y with time lag τ, $\tilde{P}(X_{}^{k},Y_{}^{k+\tau })$ is the joint probability density function of gene X(t=k) and gene Y (t=k+τ), and $\tilde{P}(X_{}^{k})$ is the marginal probability distribution functions of gene X (t=k). The higher the TDMI value is, the greater the dependence of two corresponding genes.

#### 4.1.2. Time-Delayed Maximum Information Coefficient

A maximum information coefficient (MIC), as an exploratory analysis tool, can be utilized to evaluate the relationships between hundreds of variables [49]. Compared with mutual information, MIC can reflect the relationship between two variables better [50].

A bivariate set is defined as a set where the data elements are the ordered tuples (*X*, *Y*). The maximum information gain for all the grids sized of tuple (*X*, *Y*) can be computed as [51]

(7) $$I^{*}(D,X,Y)=\max\;I(D{|}_{G}),\;$$

where G is a grid and $I(D{|}_{G})$ represents the mutual information of $D{|}_{G}$.

M(D) is the characteristics matrix of *D*, which could be represented as:

(8) $$M{\left(D\right)}_{X,Y}=\frac{I^{*}(D,X,Y)}{\log(\min(X,Y))}.\;$$

$\max(M{\left(D\right)}_{X,Y})$ is the MIC of two genes *X* and *Y*. Ideally, if two genes have no regulatory relationship, their MIC value should be about 0.

In order to reflect the time-delayed relationship between two genes, a time-delayed maximum information coefficient (TDMIC) with time lag τ is proposed, which is calculated as follows:

(9) $$\max(M{\left(D,\tau \right)}_{X,Y})=\max(\frac{I^{*}(D,X^{t},Y^{t+\tau })}{\log(\min(X^{t},Y^{t+\tau }))}).\;$$

#### 4.1.3. Time-Delayed Correlation Coefficient (TDCC)

The correlation coefficients of all genes for each time lag τ are obtained by using the time-delayed correlation coefficient (TDCC). The formula is as follows:

(10) $$C_{XY}(\tau )=\frac{\sum_{k=1}^{T}\left(X(k+\tau )-\bar{X})(Y(k+\tau )-\bar{Y}\right)}{\sqrt{\sum_{k=1}^{T}{\left(X(k)-\bar{X}\right)}^{2}}\sqrt{\sum_{k=1}^{T}{\left(Y(k)-\bar{Y}\right)}^{2}}}\;$$

where $\bar{X}=\frac{1}{T}\sum_{k=1}^{T}X(k)$, T is the number of time points, and τ is the time lag between gene X and gene Y.

#### 4.1.4. Our Proposed Scoring Method

**Figure 14:**
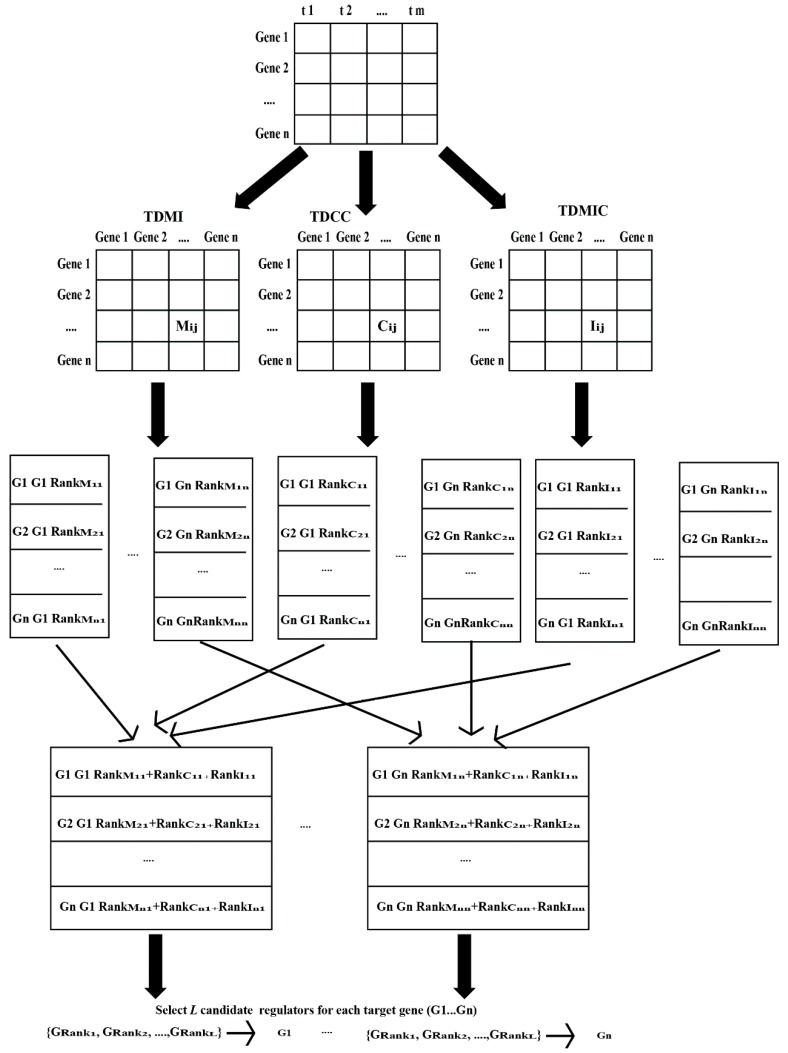
The flowchart of our proposed scoring method.

**Figure 15:**
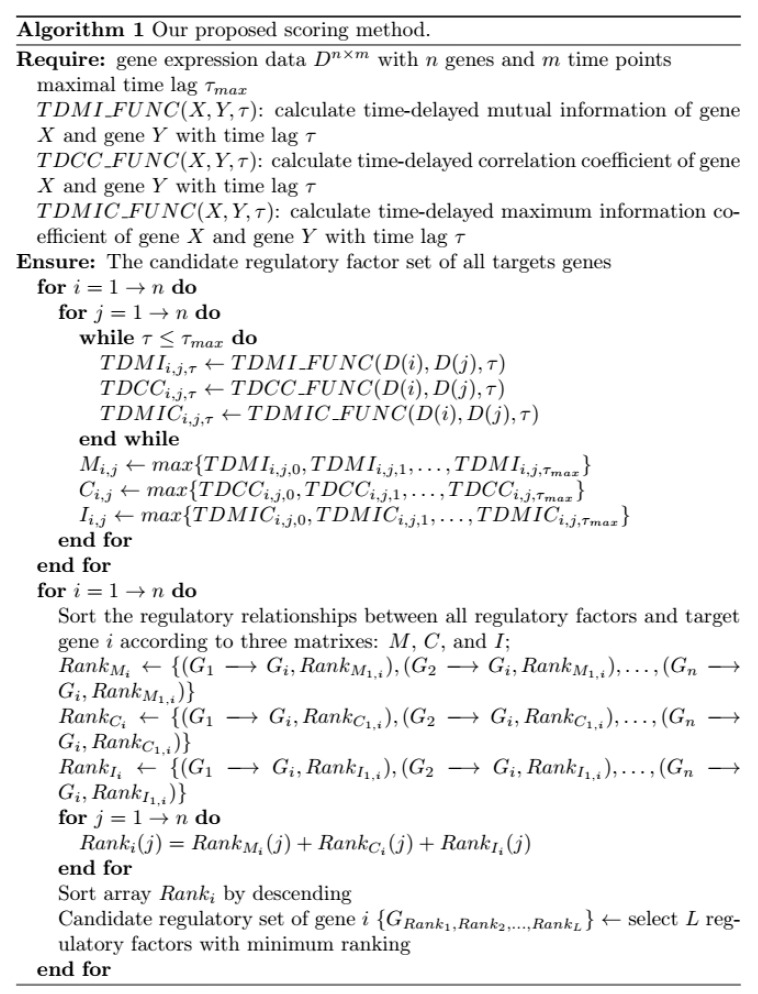
The pseudo code of our proposed scoring method.

In order to measure the time-delayed regulatory relationships accurately, a novel hybrid scoring method based on time-delayed mutual information, time-delayed maximum information coefficient and time-delayed correlation coefficient is proposed. Suppose that a time-delayed gene regulatory network contains *n* genes and gene expression time series dataset includes *m* time points. The flowchart and pseudo code of our proposed scoring method are depicted in Figure 14 and Figure 15, respectively.

### 4.2. Complex-Valued Flexible Neural Tree

Flexible neural tree (FNT) model was initially introduced by Prof. Chen in 2005 [52]. Because of its automatic features extraction, cross layer connections and flexible activation functions, the FNT model performed better than many classical neural networks and has been applied widely for solving forecasting and classification problems [53,54,55,56,57]. Due to fact that a complex-valued neural network is more flexible and functional, a complex-valued flexible neural tree (CVFNT) model, as the extension of a real-valued FNT model, is proposed to infer the time-delayed regulations in GRN [58,59,60]. A tree-structural based encoding method with a specific instruction set is selected for representing a CVFNT model. In order to create the CVFNT models randomly, two operator sets (function set *F* and terminal set *T*) are defined in advance:

(11) $$\left\{ \begin{array}{l} F=\{{+}_{2},{+}_{3},\ldots ,+N\}\\ T=\{z_{1},z_{2},\ldots ,z_{n}\} \end{array}\right.,\;$$

where ${+}_{i}$ represents the summation of *i* variables. $z_{i}$ is a terminal nodes’ instruction and takes no operands (i=1,2,…,n, $z_{i}\in C^{n}$, $z_{i}=x_{i}+jy_{i}$ and *j* stands for the value of $\sqrt{-1}$).

**Figure 16:**
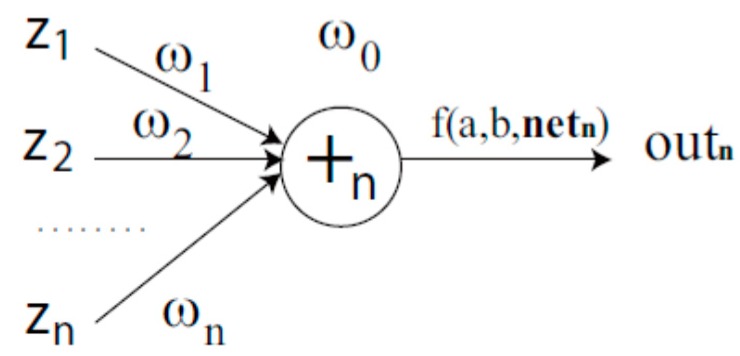
A complex-valued flexible neuron operator.

In the process of generating a CVFNT model randomly, the function and terminal operators are selected from the instruction sets *F* and *T*. If a terminal instruction $z_{i}$ is selected, the branch is terminated. If a function instruction ${+}_{n}$ is selected, *n* leaf nodes and complex-valued weights ($w_{1},w_{2},\ldots w_{n}$) are randomly created. The output of a non-terminal node can be computed as a complex-valued flexible neuron (CVFN) model (see Figure 16).

The output of a CVFN ${+}_{n}$ can be calculated as follows. The total excitation of ${+}_{n}$ is

(12) $$net_{n}=w_{0}+\sum_{j=1}^{n}w_{j}z_{j},\;$$

where $w_{0}$ is the threshold value and $z_{j}(j=1,2,\ldots ,n)$ is the complex-valued input of CVFN operator. The output of the node ${+}_{n}$ is then calculated by a complex-valued activation function (CVAF). Generally, there are three kinds of CVAFs: complex-valued Elliot function, Gaussian function and Sigmoid function, which are described as follows:

(13) $$out_{n}=f(a,r,net_{n})=\frac{net_{n}}{a+\frac{1}{r}\left|net_{n}\right|},$$

(14) $$out_{n}=f(c,\sigma ,net_{n})=e^{-\frac{{\left(net_{n}-c\right)}^{H}(net_{n}-c)}{\sigma^{2}}},$$

(15) outn=f(netn)=11+e−Re(netn)+j11+e−Im(netn),

where *f*(*·*) is CVAF and the output is complex-valued. *a*, *r* and σ are real variables, *c* is complex-valued and *|net_n_|* is the modulus of complex-valued *net_n_*. In this paper, we choose complex-valued Elliot function as a CVAF of the CVFNT model.

**Figure 17:**
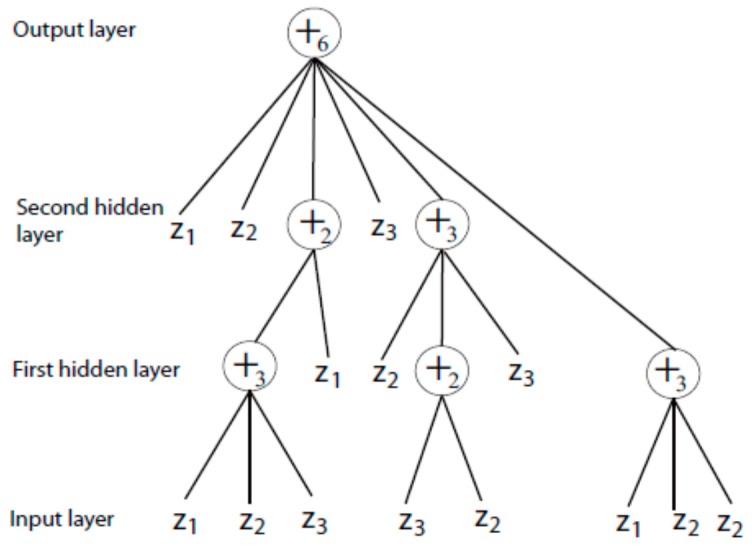
A typical CVFNT model with function set $F=\{{+}_{2},{+}_{3},{+}_{4},{+}_{5},{+}_{6}\}$, and terminal instruction set $T=\{z_{1},z_{2},z_{3}\}$.

A typical CVFNT model is described in Figure 17. The total output of CVFNT model can be calculated from left to right by a depth-first search method.

### 4.3. Flowchart of Our Method

**Figure 18:**
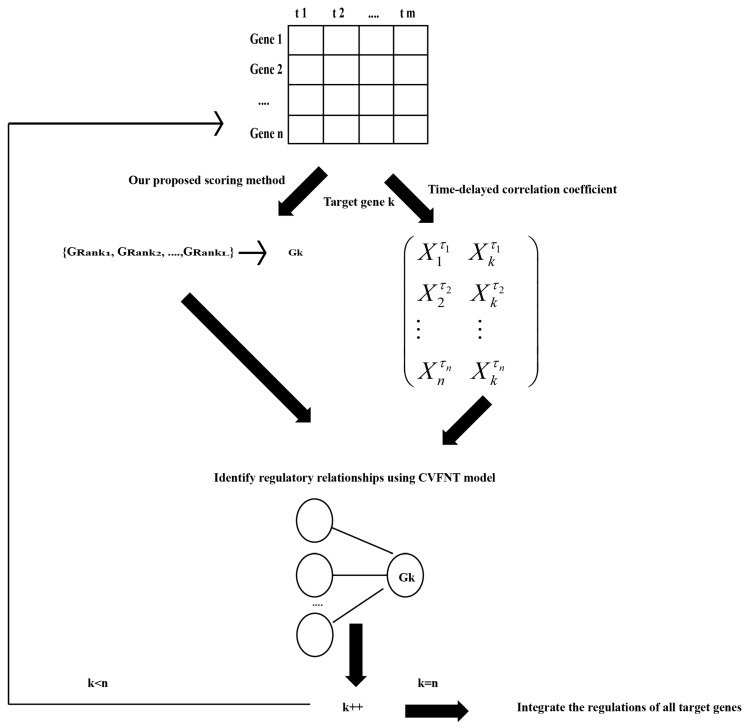
The flowchart of an HSCVFNT algorithm.

The flowchart of our HSCVFNT algorithm is described in Figure 18.

**Figure 19:**
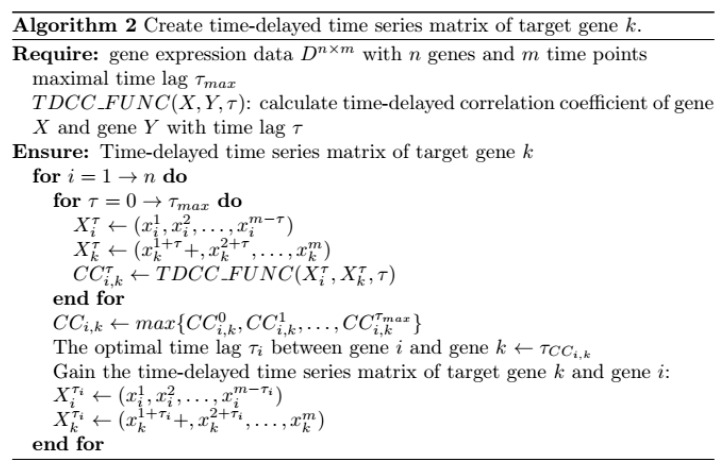
The pseudo code of creating time-delayed time series matrix of target gene *k*.

(1) Let gene expression data to be $D=\{X_{1},X_{2},\ldots ,X_{n}\}$, where $X_{i}=\{x_{i}^{1},x_{i}^{2},\ldots ,x_{i}^{m}\}$. Maximal time lag $\tau_{\max}$ and the number of candidate regulatory factors L are defined in advance.
(2) For target gene k, our proposed scoring method is utilized to select a candidate regulatory factor set $\left\{G_{Rank_{1}},G_{Rank_{1}},\ldots ,G_{Rank_{L}}\right\}$ with data D, which is described in detail in Section 4.1.4.
(3) For target gene k, its time-delayed time series matrix is created. The algorithm is described in Figure 19. Time-delayed time series matrix between gene k and its regulatory factors is listed in Equation (16):
  (16) $$\left(\begin{array}{l} X_{1}^{\tau_{1}}\;\;X_{k}^{\tau_{1}}\;\\ X_{2}^{\tau_{2}}\;\;X_{k}^{\tau_{2}}\;\\ \vdots \;\;\;\;\;\;\;\;\vdots \;\;\;\;\;\;\\ X_{n}^{\tau_{n}}\;\;X_{k}^{\tau_{n}}\;\; \end{array}\right),$$
  where $\tau_{i}$ is the optimal time lag between gene i and gene k, $X_{i}^{\tau_{i}}=(x_{i}^{1},x_{i}^{2},\ldots ,x_{i}^{m-\tau_{i}})$, and $X_{k}^{\tau_{i}}=(x_{k}^{1+\tau_{i}},x_{k}^{2+\tau_{i}},\ldots ,x_{k}^{m})$.
(4) According to the candidate regulatory set and time-delayed time series matrix of gene *k*, the CVFNT model is utilized to identify the time-delayed regulatory relationships. The gene expression data of candidate regulatory factors are utilized as input data. The gene expression data of target gene *k* are utilized as output data. The optimization process of the CVFNT model is described as follows:
  (a) Initialize the population, containing the structure, real-valued and complex-valued parameters of the CVFNT model.
  (b) All individuals in the population are evaluated, using root mean squared error (*RMSE*):
  (17) $$RMSE=\sqrt{\frac{1}{N}\sum_{i=1}^{N}{\left(y_{actual}^{i}-y_{predicted}^{i}\right)}^{2}},$$
  where N is the number of data points, $y_{actual}^{i}$ is the actual output of *i*-th time point and $y_{predicted}^{i}$ is the predicted output of the *i*-th time point.
  If the optimal CVFNT model is found or the maximum number of iterations is reached, the optimization process is over; otherwise, go to (c).
  (c) Structure is optimized using three operators: selection, crossover and mutation, which are introduced in Ref [61]. At some iterations, a certain proportion of the individuals is selected to optimize the parameters using a bat algorithm, which is introduced in detail in Refs [62,63]. Go to (b):
(5) According to the structure of the optimized CVFNT model, input genes are seen as the regulatory factors. Obtain the regulations between target gene *k* and its regulatory factors.
(6) k=k+1. If k≤n, go to (2); otherwise, the regulations of all target genes are integrated in order to infer a time-delayed gene regulatory network.

## 5. Conclusions

In order to infer a gene regulatory network accurately, a novel algorithm, namely HSCVFNT, is proposed to infer a gene regulatory network with time-delayed regulations. In the HSCVFNT algorithm, the novel scoring method is proposed to reduce the redundant regulatory relationships and obtain the candidate regulatory factor set of each target gene. The CVFNT model is utilized to infer the time-delayed regulations with a time-delayed gene expression matrix. Three real time-series expression datasets from SOS network and IRMA network are utilized to evaluate the performance of the HSCVFNT algorithm.

The results on the SOS network show that our HSCVFNT method significantly outperforms the three other state-of-the-art methods (BN, S-system and TDSS). The results on the IRMA network reveal that the HSCVFNT algorithm performs better than HRNN, MMHO-DBN, TDARACNE, TDLASSO, DBmcmc, and DBN-ZC. We also investigate the effect of a time-delayed factor and the number of candidate regulatory factors on the HSCVFNT algorithm for inferring GRN. The experiment results show that a HSCVFNT algorithm with a time-delayed factor could identify a gene regulatory network more accurately. Different numbers of candidate regulatory factors have a great influence on the performance of the algorithm. The comparison results show that about 30% of the number of genes is more appropriate.

In the future, we will apply the proposed algorithm for inferring a large-scale real gene regulatory network and discovering some meaningful relationships.

## Figures and Tables

**Table 1:** Comparison of four methods for SOS network inference.

Methods	Sensitivity	Specificity	F-Score
**HSCVFNT**	0.857	1.00	0.923
**BN**	0.5714	0.931	0.6154
**S-system**	0.857	0.862	0.706
**TDSS**	1.00	0.896	0.875

**Table 2:** Comparison of seven methods for IRMA network inference with on dataset.

Method	Precision	Sensitivity	F-Score
**HSCVFNT**	0.857	0.75	0.8
**HRNN**	0.667	0.75	0.70
**MMHO-DBN**	1.0000	0.5000	0.6667
**TDARACNE**	0.7142	0.6250	0.6667
**TDLASSO**	0.4000	0.2500	0.3077
**DBmcmc**	0.5000	0.2500	0.3333
**DBN-ZC**	0.6000	0.3750	0.4615

**Table 3:** Comparison of five methods for IRMA network inference with off dataset.

Method	Precision	Sensitivity	F-Score
**HSCVFNT**	0.625	0.625	0.625
**MMHO-DBN**	0.6667	0.2500	0.363
**TDARACNE**	0.5000	0.1250	0.2000
**TDLASSO**	0.2500	0.1250	0.1667
**DBmcmc**	0.1700	0.1200	0.1407

**Table 4:** Comparison of four methods for SOS network inference.

Method	Sensitivity		Specificity		F-Score		Hit Ratio
	mean	SD	mean	SD	mean	SD	
**HSCVFNT** **-BA**	0.8286	0.0602	0.9897	0.017	0.8856	0.0541	65%
**HSCVFNT** **-PSO**	0.8143	0.069	0.9793	0.029	0.8589	0.0772	50%
**HSCVFNT-DE**	0.8286	0.0614	0.9655	0.039	0.8449	0.0834	45%
**HSCVFNT-GA**	0.7714	0.0999	0.9517	0.037	0.7845	0.0995	35%

**Table 5:** Comparison of four methods for IRMA network inference with on dataset.

Method	Precision		Sensitivity		F-Score		Hit Ratio
	mean	SD	mean	SD	mean	SD	
**HSCVFNT** **-BA**	0.817	0.064	0.736	0.042	0.774	0.045	55%
**HSCVFNT** **-PSO**	0.796	0.096	0.694	0.091	0.740	0.086	45%
**HSCVFNT-DE**	0.788	0.117	0.702	0.095	0.737	0.096	55%
**HSCVFNT-GA**	0.791	0.072	0.681	0.091	0.730	0.076	40%

**Table 6:** Comparison of four methods for IRMA network inference with off dataset.

Method	Precision		Sensitivity		F-Score		Hit Ratio
	mean	SD	mean	SD	mean	SD	
**HSCVFNT** **-BA**	0.569	0.066	0.612	0.040	0.589	0.047	50%
**HSCVFNT** **-PSO**	0.539	0.064	0.575	0.087	0.553	0.066	40%
**HSCVFNT-DE**	0.545	0.067	0.600	0.053	0.569	0.051	35%
**HSCVFNT** **-GA**	0.516	0.059	0.588	0.060	0.547	0.048	20%

## Abbreviations

SOS	Save Our Soul
TDSS	time-delayed S-system
GRN	gene regulatory network
CVFNT	complex-valued flexible neural tree
TP	True Positive
FP	False Positive
FN	False Negative
TN	True Negative
